# COVID-19 recommender system based on an annotated multilingual corpus

**DOI:** 10.5808/gi.21008

**Published:** 2021-09-30

**Authors:** Márcia Barros, Pedro Ruas, Diana Sousa, Ali Haider Bangash, Francisco M. Couto

**Affiliations:** 1 Large-Scale Informatics Systems Laboratory, Faculdade de Ciências, Universidade de Lisboa, 1749-016 Lisboa, Portugal; 2 Center for Astrophysics and Gravitation, Faculdade de Ciências, Universidade de Lisboa, 1749-016 Lisboa, Portugal; 3 Shifa College of Medicine, Shifa Tameer-e-Millat University, Islamabad 46000, Pakistan; 4 Working Group 3, COST Action EVidence-Based RESearch (EVBRES), Western Norway University of Applied Sciences, Inndalsveien 28, 5063 Bergen, Norway

**Keywords:** COVID-19, entity extraction, recommendation, relation extraction, text mining

## Abstract

Tracking the most recent advances in Coronavirus disease 2019 (COVID-19)‒related research is essential, given the disease's novelty and its impact on society. However, with the publication pace speeding up, researchers and clinicians require automatic approaches to keep up with the incoming information regarding this disease. A solution to this problem requires the development of text mining pipelines; the efficiency of which strongly depends on the availability of curated corpora. However, there is a lack of COVID-19‒related corpora, even more, if considering other languages besides English. This project's main contribution was the annotation of a multilingual parallel corpus and the generation of a recommendation dataset (EN-PT and EN-ES) regarding relevant entities, their relations, and recommendation, providing this resource to the community to improve the text mining research on COVID-19‒related literature. This work was developed during the 7th Biomedical Linked Annotation Hackathon (BLAH7).

## Introduction

Coronavirus disease 2019 (COVID-19) pandemic took the world by surprise due to its impact on global public health. The scientific community, sensing the danger posed by this global health emergency, was quick to join hands in a bid to mitigate its effects. Natural language processing and machine learning (ML) research also focused in the quest of curbing the morbidity and mortality associated with the pandemic, being LitCovid [1] and CORD-19 [2] good examples of this effort. These resources are massive databases of scientific literature generated throughout the world pertinent to the COVID-19 pandemic— the characteristics of its causative organism (severe acute respiratory syndrome coronavirus 2), pathophysiology of the ailment as well as preventive measures that are suggested to be employed.

Recommender systems (RS) are tools for predicting the best items of interest for the users of a system, being mostly based on the past interests of the users. The interests of the users are usually collected through explicit or implicit feedback, for example, using a 5 stars system or the products opened by the users, respectively. The feedback is then used to create recommendation datasets of <user,item,rating>, useful for developing and evaluating recommendation algorithms. The main approaches used in RS are collaborative-filtering, which uses the similarity between the ratings of the users and its only dependent on the feedback of the users, and content-based, which uses the similarity/relation between the items. RS have been widely used for recommending movies, books, or e-commerce, achieving excellent results. In scientific fields, such as Health and Life Sciences, RS began to be used with the goal of helping health staff and researchers, for example, by recommending drugs to a researcher based on the drugs that she/he already had interest in. The major challenge for RS in scientific fields is the lack of open source recommendation datasets. Some alternatives have been developed, one in particular called LIBRETTI, which uses the scientific literature for creating such datasets [3].

Earlier on, it was realized that a massive resource of literature surely would come in handy while developing management protocols and RS by training ML models. Therefore, efforts have been made to create such pipelines and to fit them onto ML models that allow recommendation [4]. However, since medical literature has its own specific linguistic characteristics and that is fairly more complex than generic text, it was observed that semi-automatic annotation is critical in creating a richer constellation of medical data that can be used for superior training of recommender ML models. Moreover, a large portion of health related text is normally generated in the native language, so text mining tools should also be able to process multilingual corpora.

Therefore, the goals of the present project were to retrieve COVID-19 related documents, to automatically annotate them with entities and relations, generate recommendation datasets of scientific entities, and to manually validate a sample of the obtained annotations. The recommendation datasets are then used to develop new recommendation algorithms in the field of COVID-19.

The contributions of the present work are an automatic pipeline for document retrieval, entity and relation extraction, and recommendation, as well as a set of multilingual parallel datasets (English/Portuguese/Spanish) related with COVID-19 that allows the evaluation of Named Entity Recognition/Linking, Relation Extraction (RE), and Recommendation Systems. We also developed a new recommendation algorithm, called Relation Recommendation Algorithm (RelRA), and conducted preliminary tests with it.

## Methodology

**Fig. 1.**
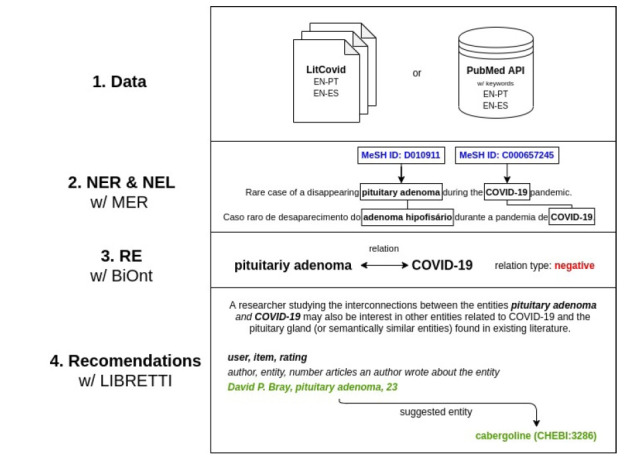
Pipeline with the tools used at each stage with an example retrieved from article PMID:33220478. NER, Named Entity Recognition; NEL, Named Entity Linking; RE, Relation Extraction; LIBRETTI, LIterature Based RecommEndaTion of scienTific Items; COVID-19, coronavirus disease 2019.

Fig. 1 presents the general workflow and the tools used throughout our work.

### Document retrieval

The first step was to retrieve COVID-19 related abstracts from PubMed repository using the Bio.Entrez package (https://biopython.org/docs/1.75/api/Bio.Entrez.html), which is part of Biopython. We used PubMed since it allows the abstract retrieval in more than one language, in our case, we needed English, Spanish, and Portuguese abstracts. Two versions of the dataset were created using different queries: *abstracts_covid_19*, which includes abstracts directly related with COVID-19 and *abstracts_large*, which includes abstracts directly and indirectly related with COVID-19. The queries used are present in Table 1.

### Entity extraction

The second step was to extract named entities present in the retrieved documents, more concretely, by performing Named Entity Recognition and Named Entity Linking. This step was accomplished by the Python implementation of MER [5], a dictionary matching system that, given a lexicon with the terms of an ontology or any knowledge base, recognizes entities in text and links them to the respective identifiers. The MER tool is light and efficient, since it does not require neither labelled data for training, as the SOTA supervised approaches usually requires (e.g., BERT) nor extensive time-consuming training. Besides, it works with any given lexicon, even if it is non-English. Consequently, we considered the tool as adequate to achieve our goals in this short-term project. Biomedical entities present in Portuguese, Spanish, and English abstracts were recognized by MER and then linked to the respective DeCS (“Descritores em Ciências da Saúde”, https://decs.bvsalud.org/) term (September 2020 edition). DeCS is multilingual biomedical vocabulary built upon MeSH terminology. It includes versions in several languages, such as Portuguese, Spanish, and English, so it is suitable for our goal of creating a multilingual dataset. Almost all DeCS terms are MeSH terms, however, there exist some specific DeCS terms that do not correspond to any MeSH term (4,378 out of 34,294 terms). Additionally, recognized biomedical entities in English abstracts were linked to the following ontologies (latest edition available at January 2021): Human Disease Ontology (DO, https://disease-ontology.org/), Gene Ontology (GO, http://geneontology.org/), Human Phenotype Ontology (HPO, https://hpo.jax.org/app/), Chemical Elements of Biological Interest (ChEBI) Ontology (https://www.ebi.ac.uk/chebi/), and Coronavirus Infectious Disease Ontology (CIDO, https://github.com/CIDO-ontology/cido).

### Relation extraction

In the third step, the relation extraction module performs RE by applying the BiOnt [6] system, built to allow the extraction of relations between biomedical entities supported by ontologies (e.g., HPO and GO). We opted for BiOnt due to its unique use of added external knowledge in the form of biomedical ontologies to potentiate RE. Thus, instead of relying on just the training data for the learning process as most RE systems, BiOnt also adds the ancestry information to each entity in a candidate pair by matching it to an ontology term.

In new data, the BiOnt system can identify relations between different and the same type of biomedical entities, such as diseases and human phenotypes, provided we use the pre-trained models trained on available training data. Using the pre-trained models available, we extracted relations between ChEBI and DO entities and between GO and HPO entities for this project. BiOnt does not make available pre-trained models for all other combinations that we could apply to our entities. We only considered relations in English abstracts since both Portuguese and Spanish abstracts only had annotated MeSH terms, for which we did not have pre-trained models. We did not restrain the relations to sentence-level and instead considered relations within the same abstract. However, since our Portuguese and Spanish abstracts can be linked to their respective English versions, it is also possible to map the extracted relations from English to the corresponding translated abstracts.

### Recommendation

#### Dataset creation

In the fourth step, the goal was to create multilingual recommendation datasets. The datasets were created using a methodology called LIterature Based RecommEndaTion of scienTific Items (LIBRETTI), which consists in developing a standard <user,item,rating> dataset from research articles. The users are the authors from the scientific articles, the items are biomedical entities mentioned in the articles, and the ratings are the number of articles an author wrote about an item [3]. For this work, the input research corpus is the one retrieved in phase 1: Document retrieval, more specifically the abstracts_large collection. The items are biomedical entities recognized in phase 2, i.e., diseases from the DO, gene terms from GO, phenotypes from HPO, and chemical compounds from ChEBI.

#### RelRA

The primary goal of the RS developed in this work is to recommend entities related to the COVID-19 disease to the researchers. To that end, we developed a new recommender content-based algorithm, based on the relations between the items - RelRA. We developed RelRA during 7th Biomedical Linked Annotation Hackathon (BLAH7), and conducted the first experiments. RelRA is based on the relations between the entities extracted in phase 2. It integrates phase 3: Relation extraction.Consider a user who has already rated some items, we want to know which items are suitable recommendations for this user. The goal of the algorithm is to provide a score to each unrated item in order to rank them. For that, we use the relations between the items, and the score is calculated considering how many relations an unrated item has with the items in the rated list. For this work we used a list of relations created using the method described in phase 3: Relation Extraction.

The RelRa algorithm was evaluated in the datasets EN_PT and EN_ES, created in phase 4. To avoid biases, the list of relations used for evaluating the RelRA algorithm were extracted from a sample of the CORD-19 corpus (nine thousand documents, version from 2020-03-13) with research articles completely different from those used to create the recommendation datasets.

#### Evaluation

For the evaluation, we tested the RelRa algorithm against a random algorithm. We used a cross-validation strategy, with 80% for the training set and 20% for the test set. For the evaluation the datasets were filtered, thus each user had at least 20 items rated. The evaluation metrics are Precision, Recall, and Mean Reciprocal Ranking (MRR) [7].

### Manual validation at BLAH7

For manual validation of the obtained annotations, we randomly selected a sample of 40 English (20) and Portuguese (20) abstracts belonging to *abstracts_covid_19* set with entity annotations and, in the case of the English abstracts, also with relation annotations. During BLAH7, annotations were uploaded to PubAnnotation (http://pubannotation.org/) and 4 participants were responsible for the correction of the existing entity and relation annotations, but also for the addition of new annotations, if deemed necessary.

## Results and Discussion

Statistics about the retrieved documents and the dimensions of the recommendations datasets created from each corpus are available in (Table 2). As expected, the abstracts_large datasets have a much higher number of documents, both in PT and ES, than the limited abstracts_COVID_19. Therefore, the recommendations datasets created from the abstracts_large corpus have as well a higher number of users, items and ratings.

**Fig. 2.**
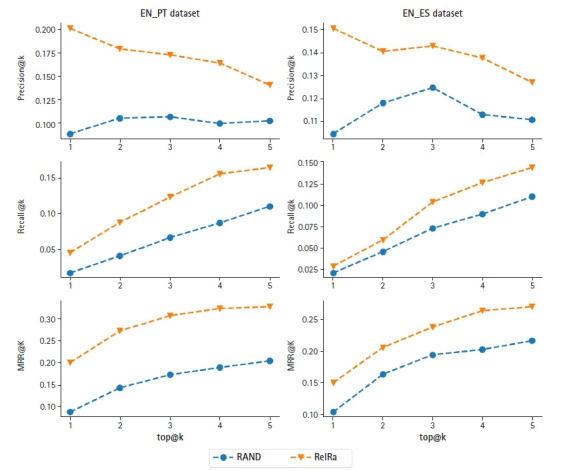
Results for RelRa vs. RAND, for precision, recall and MRR, for EN_PT and EN_ES datasets. RelRA, Relation Recommendation Algorithm; RAND, Random; MRR, Mean Reciprocal Ranking; EN_PT, created from abstracts in English (“EN”) and Portuguese (“PT”); EN_ES, created from abstracts in English (“EN”) and Spanish (“ES”).

The results of the algorithms RelRA and Random (RAND) for the datasets EN_PT and EN_ES are presented in Fig. 2, respectively, for the evaluation metrics of Precision, Recall and MRR, for the top@5 ranked results.

Fig. 2 shows that RelRa achieved better results for all the evaluation metrics when compared with a random recommendation of the items, for both EN_PT and EN_ES datasets. RelRa seems to have great potential for the recommendation of scientific entities based on their relations, however, the number of documents and relations needs to be improved for further testing.

**Fig. 3.**
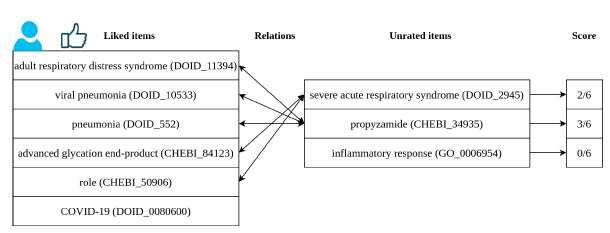
Example of recommendation using the RelRA algorithm. RelRA, Relation Recommendation Algorithm; COVID-19, coronavirus disease 2019.

Fig. 3 shows an example of recommendation. The user represented had interest in six different entities. We have a list of three entities that we wish to know if are suitable for recommendation to this user. Using RelRA, we find the relations between the list of liked items and these unknown items. Severe acute respiratory syndrome (DOID_2945) is related to two items in the liked list, thus it has a score of 2/6. Propyzamide (CHEBI_34935) is related to three entities in the liked list, achieving a score of 3/6. Inflammatory response (GO_0006954) does not have any relation with the items liked by this user. Severe acute respiratory syndrome (DOID_2945) and Propyzamide (CHEBI_34935) would be recommended to this user.

These are the first tests with RelRA, which was specially developed during BLAH7.

### Manual validation at BLAH7

Table 3 presents the results for the manual validation stage at BLAH7. After the validation process, for the 40 documents, we retrieved more entities, more relations, and with better quality by discarding illy annotated entities and relations. Further, by reaching a consensus between the four annotators, we increased our datasets’ quality by adding even more annotations. These datasets are available in the PubAnnotation (http://pubannotation.org/collections/LASIGE:%20Annotating%20a%20multilingual%20COVID-19-related%20corpus%20for%20BLAH7) platform (in their original and consensus format).

## Conclusion

Our goals for the present project were to retrieve COVID-19 related documents, to automatically annotate them with entities and relations, generate recommendation datasets of scientific entities, and to manually validate a sample of the obtained annotations.

We were able to create an automatic pipeline for document retrieval, entity and relation extraction, and recommendation, as well as a set of multilingual parallel datasets (English/Portuguese/Spanish) related with COVID-19 that allows the evaluation of Named Entity Recognition/Linking, RE, and Recommendation Systems. Further, we partially manually validated our datasets using the PubAnnotation platform.

For future work, the manual validation of the annotations could be improved, more concretely, by leveraging crowdsourcing platforms to recruit a large number of annotators [8]. Besides, due to time constraints, we were not able to manually validate the annotations present in EN_ES datasets during BLAH7, so future work could accomplish this.

## Figures and Tables

**Table 1.** Queries used for document retrieval

Set	English query	Portuguese query	Spanish query
abstracts_covid_19	*covid-19 AND English [LANG]*	*AND English [LANG] AND Portuguese [LANG]*	*AND English [LANG] AND Spanish [LANG]*
abstracts_large	*2019 Novel Coronavirus Disease OR 2019 Novel Coronavirus Infection OR 2019-nCoV Disease OR 2019-nCoV Infection OR COVID-19 Pandemic OR COVID-19 Pandemics OR COVID-19 Virus Disease OR COVID-19 Virus OR Infection OR COVID19 OR Coronavirus Disease 2019 OR Coronavirus Disease-19 OR SARS Coronavirus 2 Infection OR SARS-CoV-2 Infection AND English [LANG]*	*2019 Novel Coronavirus Disease OR 2019 Novel Coronavirus Infection OR 2019-nCoV Disease OR 2019-nCoV Infection OR COVID-19 Pandemic OR COVID-19 Pandemics OR COVID-19 Virus Disease OR COVID-19 Virus OR Infection OR COVID19 OR Coronavirus Disease 2019 OR Coronavirus Disease-19 OR SARS Coronavirus 2 Infection OR SARS-CoV-2 Infection AND English [LANG] AND Portuguese [LANG]*	*2019 Novel Coronavirus Disease OR 2019 Novel Coronavirus Infection OR 2019-nCoV Disease OR 2019-nCoV Infection OR COVID-19 Pandemic OR COVID-19 Pandemics OR COVID-19 Virus Disease OR COVID-19 Virus OR Infection OR COVID19 OR Coronavirus Disease 2019 OR Coronavirus Disease-19 OR SARS Coronavirus 2 Infection OR SARS-CoV-2 Infection AND English [LANG] AND Spanish [LANG]*

**Table 2.** Number of documents in each version of the dataset, and respective dimensions of the recommendation datasets

Set	Languages	Abstracts	nUsers	nItems	nRatings
abstracts_covid_19	EN_PT	80	1,750	1,507	36,614
	EN_ES	53	1,744	669	14,920
abstracts_large	EN_PT	346	1,869	2,403	49,839
	EN_ES	390	1,855	1,036	20,417

EN_PT, created from abstracts in English ("EN") and Portuguese ("PT"); PT, created from abstracts in Portuguese ("PT"); EN_ES, created from abstracts in English ("EN") and Spanish ("ES"); ES, created from abstracts in Spanish ("ES"). nUsers, number of users; nItems, number of items; nRatings, number of ratings.

**Table 3.** Final counts for the 40 abstracts sample (20 English and 20 Portuguese), the mean number of each subset for the annotators/curators task, and the final consensus numbers of manual validation

Dataset		Original	Annotated/Curated	Consensus
Portuguese	Entities	245	322	354
English	Entities	493	511	607
	Relations	224	238	250
